# Feasibility randomised controlled trial of the Early Adolescent Skills for Emotions psychological intervention with young adolescents in Lebanon

**DOI:** 10.1186/s12888-023-04571-9

**Published:** 2023-03-01

**Authors:** Felicity L. Brown, Karine Taha, Frederik Steen, Jeremy Kane, Aviva Gillman, May Aoun, Aiysha Malik, Richard Bryant, Marit Sijbrandij, Rabih El Chammay, Chiara Servili, Mark van Ommeren, Aemal Akhtar, Edwina Zoghbi, EASE Intervention Development, Training Team, Katie S. Dawson, Sarah Watts, Maha Ghatasheh, May Aoun, Aiysha Malik, Felicity L. Brown, Mark J. D. Jordans, on behalf of the STRENGTHS Consortium, Ceren Acarturk, Aemal Akhtar, Ibrahim Akinçi, Ahmed Bawaneh, Martha Bird, Felicity L. Brown, Richard Bryant, Sebastian Burchert, Pim Cuijpers, Anne de Graaff, Annelieke Drogendijk, Daniela Fuhr, Jonas Maria Hessling, Zeynep Ilkkursun, Mark J. D. Jordans, Christine Knaevelsrud, Gülşah Kurt, David McDaid, Saara Martinmäki, Cansu Mirzanlı, Trudy Mooren, Naser Morina, A.-La Park, Monique Pfaltz, Bayard Roberts, Matthis Schick, Ulrich Schnyder, Marit Sijbrandij, Egbert Sondorp, Julia Spaaij, Frederik Steen, Karine Taha, Peter Ventevogel, Claire Whitney, Nana Wiedemann, Aniek Woodward

**Affiliations:** 1 Research and Development Department, War Child Holland, Amsterdam, The Netherlands; 2 Amsterdam Institute of Social Science Research, University of Amsterdam, Amsterdam, the Netherlands; 3 War Child Lebanon, Beirut, Lebanon; 4 Mailman School of Public Health, Columbia University, New York, USA; 5 Kings College London, London, UK; 6 Department of Mental Health and Substance Abuse, World Health Organization, Geneva, Switzerland; 7 School of Psychology, University of New South Wales, Sydney, NSW Australia; 8 Clinical, Neuro and Developmental Psychology, VU University, Amsterdam, The Netherlands; 9 Ministry of Public Health, Beirut, Lebanon; 10 Department of Psychiatry, Faculty of Medicine, Saint Joseph University, Beirut, Lebanon; 11 Division of Insurance Medicine, Department of Clinical Neuroscience, Karolinska Institutet, Stockholm, Sweden; 12 World Health Organization, Beirut, Lebanon

**Keywords:** Feasibility trial, Psychological intervention, Refugee mental health, Young adolescents

## Abstract

**Background:**

Globally, there is a vast mental health treatment gap, whereby the majority of adolescents living in low- and middle-income countries requiring mental health services, do not have access to adequate care. To improve access, the World Health Organization (WHO) developed a range of interventions, designed to be low-cost and delivered by non-specialists. We conducted a two-arm, individually randomised group treatment feasibility trial of a new WHO group intervention for young adolescents with emotional distress (‘Early Adolescent Skills for Emotions’; EASE) in Lebanon.

**Method:**

The aim of this study was to determine the feasibility of the intervention and study procedures. Adolescents aged 10 to 14 years were eligible to take part if they scored above a validated cut-off on the Child Psychosocial Distress Screener. Participants were randomized to EASE or enhanced treatment as usual (ETAU) control using a 1:1 ratio. EASE consisted of seven group sessions with adolescents and three sessions with caregivers. ETAU consisted of a single brief psychoeducation home visit. Child and caregiver outcomes were measured by blind assessors at baseline, endline (8 weeks post-randomisation), and three month follow-up (20 weeks post-randomisation), with the primary outcome measure being child psychological symptoms on the Pediatric Symptom Checklist. Qualitative interviews were conducted with adolescents (*n* = 13*)*, caregivers (*n* = 17), facilitators (*n* = 6), trainers (*n* = 3), and outreach staff (*n* = 1) at endline to assess barriers and facilitators related to the feasibility and delivery of EASE and study procedures.

**Results:**

Of 154 adolescents screened, 67 (43%) were eligible, completed baseline, and were randomized. Sixty adolescents (90%) completed endline assessments (31 EASE, 29 ETAU), and fifty-nine (88%) completed three-month assessments (29 EASE, 30 ETAU). Qualitatively, participants provided overall positive feedback about the intervention. Several challenges and suggestions for improvement were raised around logistics, intervention content, and acceptability of assessment measures. Implementation data highlighted challenges with intervention uptake and attendance. Outcome measures generally had strong psychometric properties (range: α = 0.77 to α = 87), however did not demonstrate change over time in either group.

**Conclusions:**

The EASE intervention and study procedures are acceptable and feasible for implementation with vulnerable adolescents in Lebanon, however several improvements are necessary prior to full-scale evaluation.

**Trial registration:**

#ISRCTN60799626, retrospectively registered on 04/10/2022.

## Background

At the end of 2021, almost 90 million people were forcibly displaced; 27 million refugees, and more than 40% children [1]. Throughout 2022, this figure has increased to more than 100 million [1]. Armed conflict is a major driver of displacement and has a huge impact on mental health and wellbeing of populations. It is estimated that one in five individuals in conflict-affected areas will have a diagnosable mental disorder, with the burden of disease of common mental disorders more than five times the global average [2]. Children and young people frequently experience additional stressors and barriers to healthy development before, during and after conflict, including displacement, poverty, education interruptions, increased family and community violence, and child protection risks [3], placing them at greater risk of mental health problems [4].

The majority (83%) of refugees are hosted in Low and Middle Income Countries (LMICs) [1], where host communities face similar social determinants of poor mental health, and health systems are drastically under-resourced to deal with the increased demands [5]. Therefore, in these settings, a vast treatment gap exists, whereby the majority of individuals needing mental health treatment do not receive minimally adequate care [6, 7]. This is especially the case for young people where there are significant system-level barriers to providing necessary support, including scarcity of mental health professionals [8] and inadequate funding for mental health, especially for children and adolescents [9, 10].

To overcome this treatment gap, effort has shifted towards scalable psychological interventions that can be delivered by trained and supervised non-specialist providers, following a task-sharing approach [6]. International guidelines [11] recommend delivering focused, non-specialist interventions for individuals experiencing distress, reserving specialist care for more severe and complex cases. Several such interventions for adults have been developed and evaluated across different settings with good effectiveness and feasibility, including the World Health Organization’s (WHO) (Group-) Problem Management Plus (PM +) [12–15]. Yet similar evidence-based brief non-specialist interventions specifically for children and adolescents remain scarce.

Although WHO guidelines recommend psychological interventions such as cognitive behavioural therapy for adolescents with emotional disorders [16], available evidence for effectiveness of interventions with children and adolescents in humanitarian settings is mixed, with a strong focus on treatments for posttraumatic stress disorder (PTSD) [17]. A recent individual patient data meta-analysis found significant effects of focused psychosocial interventions in reducing symptoms of PTSD, and improving functioning and strengths including coping, hope, and social support [18]. However, no significant effects were seen for reduction of depression and anxiety, and effects on other outcomes were stronger in older adolescents (15–18 years old). Similarly, an umbrella review of systematic reviews found only suggestive evidence of effectiveness for psychosocial interventions for children in humanitarian settings in reducing PTSD, and for general child populations in reducing disruptive behavior, with weak or no evidence for other outcomes [19].

WHO recently developed the Early Adolescent Skills for Emotions (EASE) intervention to reduce psychological distress for adolescents aged 10–14 years who are living in adversity and experiencing significant internalizing symptoms (e.g. anxiety or depression) [20]. EASE is designed to be applicable across common mental disorders, brief, transdiagnostic, and deliverable by non-specialists, reducing the need for diagnostic specialist services which may not be available. It consists of manualized evidence-based techniques found to be most commonly included in effective psychological interventions for this age group and deemed to be safe and feasible for delivery by trained non-specialists.

We previously conducted a cultural adaptation of the EASE intervention for the context of Lebanon [21]. Following recommendations for developing complex interventions [22], we first conducted this randomized feasibility trial to inform necessary adaptations prior to the implementation of a fully powered RCT to assess effectiveness. Specifically, the aims were to:

- 1) Determine recruitment, screening, completion, and retention rates for the EASE programme and follow up assessments
- 2) Evaluate the feasibility and acceptability of intervention delivery of EASE by trained non-specialists
- 3) Assess feasibility of outcome measures and their psychometric properties, and explore trends in changes from baseline to endline within each group, and between groups
- 4) Assess feasibility and safety of trial procedures such as randomization, blinding of assessors, contamination, and occurrence and monitoring of adverse events

This feasibility trial and a simultaneous study in Jordan [23] were conducted in preparation for the first full-scale evaluations of EASE in Lebanon and Jordan [24, 25]. Additional studies have been conducted in Tanzania [26] and Pakistan [27].

## Methods

### Design

This study was a feasibility trial, following the intended RCT design: a two-arm individually randomised group treatment trial, with a 1:1 allocation of participants to EASE or enhanced treatment as usual (ETAU). We assessed: recruitment, screening, attendance, and retention rates of participants; fidelity and competence of facilitator delivery of the intervention; the feasibility of randomization and blinding procedures and the likelihood of contamination between treatment and control groups; psychometric properties and trends in outcome measures on a range of adolescent and caregiver outcomes at pre-intervention (“baseline”; T0), post-intervention (“endline”; approximately 8 weeks post-randomisation; T1), and 3-month follow-up (approximately 20 weeks post-randomisation; T2). At T1 we conducted a qualitative process evaluation using semi-structured interviews and focus group discussions with participants and stakeholders. Study methods and results are reported following the CONSORT guidelines on the reporting of randomized feasibility and feasibility studies [28]. The trial is retrospectively registered online (#ISRCTN60799626, date 04/10/2022), and the protocol is available from authors on request.

### Setting

Lebanon is a lower-middle-income country and globally hosts the highest number of refugees per capita, with an estimated 1.5 million Syrian plus large numbers of Palestinian refugees from a national population of 5.9 million [29]. In recent decades Lebanon has experienced prolonged internal and external conflict, leading to challenges of limited basic infrastructure, political instability, and struggling economy. It is estimated that approximately 1.4 million children in Lebanon are currently growing up with urgent unmet needs for basic services and protection [29].

We carried out the study in community centres in the North governorate of Lebanon, including urban areas in Tripoli and agricultural areas in Minieh-Dinnieh from September 2018 to July 2019. The trial was implemented by War Child, an international non-governmental organization that has been responding to the Syrian crisis in Lebanon since 2012. Ethical approval was obtained via the Ethical Review Committees at St Joseph’s University in Beirut (USJ-2017–24-bis), and the World Health Organization (ERC.0003000).

### Participants and sample size

We enrolled participants of any nationality (i.e. Lebanese, Syrian, Palestinian) if they met the following inclusion criteria: (i) aged between 10 and 14 years; (ii) resided with a caregiver who provided consent; and (iii) screened positive for psychological distress during screening. Participants were excluded if they meet any of the following criteria: (i) unaccompanied minor; (ii) caregiver was not a family member, as they were not able to provide legal consent; (iii) significant cognitive impairment or severe neurological impairments or developmental difficulties as determined by caregiver-report during screening, where this would impair their ability to participate in a group intervention; (iv) imminent risk of suicide, and; (v) currently married, due to legal consent and protection concerns. Any eligible children in a household were included in the study but siblings were randomised as a single unit to prevent allocation to different intervention arms. No power calculations were conducted as we did not aim to test for statistical differences between trial arms in this feasibility study. Instead, we aimed to enrol approximately 32 adolescents in each arm, which would result in approximately four EASE groups (approximately 6–8 adolescents per group). This was deemed sufficient to allow us to test our procedures and address the aims of this study.

### Informed consent

Informed consent from caregivers and assent from adolescents consisted of a two-step procedure: (1) to participate in screening and (2) to participate in the main study. For each step, participants were asked to complete a written consent form; for those with literacy issues, witnessed oral consent was collected.

### Recruitment, eligibility and screening

Participants were recruited using a standardized script by outreach workers from War Child, either alongside outreach for an education program, via awareness sessions, or door to door visits. After obtaining informed consent and assent, assessors administered a screening interview. Screening with adolescents consisted of the Child Psychosocial Distress Screener (CPDS; [30]) and suicide risk interview. The CPDS is a context-specific primary screening tool for psychosocial distress consisting of five child-reported items and two caregiver-reported items. It has been validated in Lebanon, with an optimal cut-off of five for identifying adolescents meeting the criteria for significant psychological distress in this setting (Brown F, Steen F, Taha K, Koppenol-Gonzalez GV, Aoun M, Bryant RA, et al: Validation of Arabic versions of the Child Psychosocial Distress Screener and Pediatric Symptom Checklist for young adolescents living in vulnerable communities in Lebanon, Under review). The suicide risk interview consisted of a set of three structured screening questions to identify imminent risk of suicide. Adolescents at imminent risk of suicide were referred to specialist support. Screening with caregivers consisted of two caregiver items on the CPDS, and four items from an adapted version of the Ten Questions instrument [31] to assess for significant developmental, neurological, or intellectual impairment that would compromise participation in the intervention. Participants were eligible for the study and invited to T0 assessments if they: i) scored above the cut off on the CPDS; (ii) were not at imminent risk of suicide; and iii) did not have significant impairments.

### Interventions

#### EASE

The EASE intervention [20] is a group psychological intervention consisting of seven 90-min group sessions for adolescents and three 120-min group sessions for their caregivers, running over approximately seven weeks. Adolescent sessions involve psychoeducation, problem solving, stress management (slow breathing), behavioural activation, and relapse prevention. The caregiver sessions are open to all adult caregivers and involve psychoeducation, active listening, quality time, praise, caregiver self-care and relapse prevention. Child groups were divided by gender, with siblings separated between groups when different genders. Caregiver sessions were scheduled so that the first occurred before the third child session, the second occurred before the fifth child session, and the third occurred before the last child session.

#### ETAU

Treatment-as-usual for adolescents living in vulnerable communities in Lebanon usually consists of very limited mental health services. Therefore, to ensure ethical response to highly vulnerable adolescents who were identified as having high distress, our comparison group was ETAU involving a single-session psychoeducation home visit. The adolescent and their caregivers were invited to the session of approximately 30-min in which they received brief feedback that the youth indicated psychological distress, along with scripted psychoeducation about (i) self-care strategies and (ii) seeking services from local health or community services offering mental health and psychosocial support services. If adolescents or caregivers remained concerned about their psychological distress, they were encouraged to seek support through local community organisations. ETAU participants were not offered EASE for the duration of their enrolment in the study. In both study arms, participants were provided with a list of hotlines they could call to find out more about other available services in their area.

#### Facilitators

Six non-specialist male and female facilitators with past experience delivering psychosocial activities in their community delivered the EASE intervention, with two facilitators per intervention group. They received nine days of training in basic counselling skills, delivery of EASE, group facilitation, self-care, and organisational trainings, including child safeguarding, suicide risk management and security briefings. Additionally, they completed a supervised practice cycle of EASE intervention (comprised of individuals without high levels of distress) and underwent an assessment of competencies to be eligible to implement the intervention. Weekly group supervision was provided by local trainers who received a training-of-trainers and had previously conducted their own EASE intervention groups. They also received training in supervisory techniques, in order to ensure protocol adherence. Trainers themselves received regular supervision (from AM, FB, or MA), to ensure treatment adherence and provide support.

ETAU facilitators were recruited using the same criteria and process as EASE facilitators. They received three days of training in delivering the scripted session, basic counselling and communication skills, and self-care. At the end of training a role-play competency assessment was conducted. Given the single session nature of ETAU, facilitators received one group supervision session mid-way through implementation of the sessions, and a group debrief and feedback session once all intervention sessions were completed.

#### Attendance, fidelity, and competency

We measured attendance of adolescents and caregivers at EASE and ETAU sessions. We considered EASE participants to be ‘treatment completers’ if adolescents attended five or more sessions of EASE. To evaluate treatment fidelity, facilitator pairs completed a session checklist at each session. We aimed to conduct observations of a sample of 10% of both ETAU and EASE sessions by trained staff members using structured checklists to score which components of each intervention session were delivered by the facilitator. In each observation of EASE sessions, intervention-specific competencies were assessed through rating the quality of delivery of each component, and core competencies were assessed through rating on four standardized items inspired by existing tools [32] (rated as ‘done well’, ‘done partially well’, or ‘needs improvement’).

### Outcome assessments

Outcome measures were selected based on psychometric properties, and appropriateness for the setting. We aimed to conduct T0 within the two weeks prior to commencing EASE or ETAU, with no more than one month between in all cases. T1 was scheduled within one to two weeks of the final EASE session and T2 at 12 weeks following T1. All instruments were translated into simple Arabic understandable to children living in Lebanon, following recommended processes [33]. All instruments were delivered via face-to-face interviews in the home or community centres by trained assessors using Kobo software on tablets. In case of travel, participants were provided with transportation or reimbursement for costs. In case participants did not attend a scheduled assessment, three attempts were made to contact them to schedule a new appointment. For full details of outcome measures, see the protocol [24].

#### Adolescent-reported outcomes

While the primary outcome of this study was feasibility of implementation, the main outcome measure of interest (planned as primary outcome in the definitive trial) was adolescent-reported psychological distress, assessed by the PSC-35 youth-report [34]. We also measured symptoms of depression using the adolescent Patient Health Questionnaire (PHQ-A; 9 items) [35] and post-traumatic stress symptoms using the Children’s Revised Impact of Event Scale (CRIES-13; 13 items) [36]. We measured impairment of daily functioning using a 9-item questionnaire developed specifically for this study using a recommended process [37]. We measured subjective wellbeing using the Warwick Edinburgh Mental Wellbeing Scale (WEMWBS; 14 items) [38].

#### Caregiver-reported outcomes

Caregivers reported on adolescent distress via the PSC-35 caregiver-report (35 items) [34]. We measured caregiver psychological distress using the Kessler Psychological Distress Scale (K6; 6 items) [39] and parenting behaviours using the Alabama Parenting Questionnaire-42 (APQ-42; 42 items) [40]. Where a caregiver had multiple adolescents in the study, the APQ-42 and K6, were only completed once by the caregiver, while the caregiver-report PSC-35 was completed separately for each child.

#### Other measures

Adolescent and family demographics were collected from caregivers (see Tables 1 and 2). To measure trauma exposure in adolescents as a demographic characteristic, we developed a 27-item traumatic events checklist to be completed by caregivers (at T0 only).

**Table 1:** Baseline Adolescent Demographic Characteristics

	**EASE (*n* = 35)**	**ETAU (*n* = 32)**
	**N (%) or Mean (SD) [Range]**	
Female	16 (45.7)	14 (43.8)
Male	19 (54.3)	18 (56.3)
Age*	11.7 (1.4) [10-14]	11.7 (1.1) [10-14]
Nationality*		
Lebanese	1 (2.9)	1 (3.1)
Syrian	34 (97.1)	31 (96.9)
Country of birth*		
Lebanon	2 (5.7)	2 (6.3)
Syria	33 (94.3)	30 (93.8)
Year family moved to Lebanon^a^		
2007–2009	1 (3.6)	0 (0.0)
2010–2012	10 (35.7)	9 (34.6)
2013–2015	13 (46.4)	11 (42.3)
2016–2018	3 (10.7)	4 (15.4)
Birth order of child*		
1^st^	8 (22.9)	15 (46.9)
2^nd^-4^th^	19 (54.3)	9 (28.1)
5^th^ or later	8 (22.9)	8 (25.0)
Currently in school?*		
Yes, formal education	17 (48.6)	22 (68.8)
Yes, non-formal education	1 (2.9)	2 (6.3)
No	16 (45.7)	8 (25.0)
Don’t know	1 (2.9)	0 (0.0)
Ever been in school *^b^		
Yes	8 (50.0)	6 (75.0)
No	8 (50.0)	2 (25.0)
Length of time spent out of school*		
0 months	10 (28.6)	12 (37.5)
1–6 months	4 (11.4)	4 (12.5)
12 + months	21 (60.0)	14 (43.8)
Full years of school completed*		
0	4 (11.4)	1 (3.1)
1–2	12 (34.3)	5 (15.6)
3–4	13 (52.0)	20 (62.5)
5–7	6 (17.1)	6 (18.8)
Brings income to the family*		
Yes	6 (17.1)	9 (28.1)
No	29 (82.9)	23 (71.9)
Helps with childcare*		
Yes	17 (48.6)	23 (71.9)
No	18 (51.4)	9 (28.1)
Participated in other programmes in past month		
Yes	7 (20.0)	5 (15.6)
No	28 (80.0)	27 (84.4)

^*^Denotes caregiver report

^a^ among those not born in Lebanon, for family rather than individual children

^b^ among those currently out of school

**Table 2:** Baseline Caregiver Demographic Characteristics

	**EASE (*n* = 28)**	**ETAU (*n* = 26)**
	**N (%) or Mean (SD) [Range]**	
Age	38.4 (7.9) [18–54]	38.4 (7.9) [27–55]
Caregiver type		
Mother	19 (67.9)	20 (76.9)
Father	7 (25.0)	5 (19.2)
Other family member	2 (7.1)	1 (3.9)
Number of children	5.7 (2.1) [2-10]	5.5 (2.8) [0–12]
Mother in the household		
Yes	26 (92.9)	25 (96.2)
No	2 (7.1)	1 (3.9)
Father in the household		
Yes	24 (85.7)	22 (84.6)
No	4 (14.3)	4 (15.4)
Other family caregiver in the household		
Yes	2 (7.1)	1 (3.9)
No	26 (92.9)	25 (96.2)
Mother’s education (among mothers in the household)		
No school	6 (23.1)	6 (23.1)
Primary school	9 (26.0)	6 (23.1)
Middle school	10 (38.5)	12 (46.2)
High school	1 (3.8)	0 (0.0)
Higher education	0 (0.0)	1 (3.9)
Father’s education (among fathers in the household)		
No school	1 (4.2)	1 (4.5)
Primary school	7 (29.2)	8 (36.4)
Middle school	16 (66.7)	11 (50.0)
High school	0 (0.0)	2 (9.1)
Other family caregiver education (among other caregivers in the household)		
Primary school	1 (50.0)	1 (100.0)
Middle school	1 (50.0)	0 (0.0)
Housing type		
Informal settlement	10 (35.7)	12 (46.2)
Rented room	5 (17.9)	1 (3.9)
Rented house	11 (39.3)	11 (42.3)
Owned property	1 (3.6)	0 (0.0)
Other	0 (0.0)	1 (3.9)
Missing	1 (3.6)	1 (3.9)
Mother’s work status (among mothers in household)		
Daily worker	2 (7.7)	5 (17.9)
Self-employed	0 (0.0)	1 (3.6)
Out of work—looking	1 (4.3)	2 (8.0)
Homemaker	23 (88.5)	17 (68.0)
Father’s work status (among fathers in household)		
Wages-full time	0 (0.0)	1 (4.5)
Wages-part time	1 (4.2)	0 (0.0)
Daily worker	16 (66.7)	14 (63.6)
Self-employed	1 (4.2)	0 (0.0)
Out of work—looking	5 (20.8)	5 (22.7)
Unable to work	1 (4.2)	2 (9.1)
Other family caregiver’s work status (among those in household)		
Wages-full time	1 (50.0)	0 (0.0)
Out of work—looking	1 (50.0)	0 (0.0)
Unable to work	0 (0.0)	1 (100.0)
Number of adult financial providers in household		
0	9 (32.1)	8 (30.8)
1–2	19 (67.9)	15 (57.7)
3 +	0 (0.0)	3 (11.5)
Number of child financial providers in household		
0	17 (60.7)	13 (50.0)
1–2	8 (28.6)	10 (38.5)
3 +	3 (10.7)	3 (11.5)
Income		
< $299	12 (42.9)	15 (57.7)
$300-$599	15 (53.6)	11 (42.3)
Missing	1 (3.6)	0 (0.0)
Participated in other programmes in past month		
Yes	0 (0.0	0 (0.0)
No	28 (100.0))	26 (100.0)

### Trial feasibility and safety

#### Randomisation

Randomisation occurred following completion of the T0 assessment. Randomisation sequences were computer generated by an independent staff member who was not involved in study implementation, using random block sizes of 2 and 4. To support practical implementation and to ensure adequate numbers in the EASE group sessions, separate randomisation sequences were created for males, females, and sibling pairs. Siblings were randomised in a separate stratum in order to maintain equal numbers between groups, and because sibling pairs could have mixed genders. Group allocations (EASE or ETAU) were recorded on pieces of paper, which were folded and placed inside sealed, numbered, opaque envelopes. The numbered envelopes were opened in sequence by the research coordinator with the allocation assigned to the corresponding adolescent on registration lists (having completed baseline assessments).

#### Blinding

We aimed to keep assessors blind to the intervention allocation of adolescents throughout the trial, while investigators, implementation staff, and participants were not blind. All staff were trained and supervised in the importance of maintaining blinding. Prior to conducting each T1 and T2 assessment, participants were instructed not to reveal their allocation to the assessor. In the case that the allocation was revealed, assessors were instructed to inform the research coordinator immediately and another assessor would be assigned to complete the assessment. At the end of each T1 and T2 assessment, assessors provided a guess as to which treatment the participant received, including any potential reasons for this guess.

#### Contamination

To assess possible contamination, participants were asked at T1 and T2 about the extent to which they shared information and materials about the treatment received with others in the community, and whether they had heard about the other treatment and materials from others. Participants were also asked if they made use of any services from the hotline list. This information was used descriptively to determine contamination.

#### Adverse events and trial management

The occurrence of specific serious adverse events (SAEs) according to War Child’s operational definition (including deaths; suicide attempts; victimization including physical, sexual and emotional abuse or neglect; serious violence; emergency psychiatric or medical hospitalisation; or serious lack of food) and adverse events (AEs; including injuries or accidents on way to or from the research activities; marked increases in suicidal thoughts; mentioning of concrete and detailed plan to commit suicide; marked increases in emotional distress; marked increases in conflicts within family or community; other violence towards staff or participants) were monitored throughout the study by field-based research and implementation teams. They were reported using structured incident report forms submitted to the lead investigators, who then reported to the Data Safety Management Committee (DSMC) and relevant ethical boards. The trial coordinator provided daily supervision and oversight to assessors during data collection. Weekly study meetings between the research coordinator and study investigators ensured adequate support for implementation, fidelity to protocol, and trial safety.

### Process evaluation

After T1 we conducted focus groups and key informant interviews with adolescents (*n* = 10*)* and caregivers (*n* = 15) completing the intervention and adolescents (*n* = 3*)* and caregivers (*n* = 2) dropping out, facilitators (*n* = 6), trainers (*n* = 3), and outreach staff (*n* = 1). Trained local assessors conducted the interviews, separate to the team conducting quantitative assessments, given that intervention allocation of families would be revealed in these interviews. Interviews followed semi-structured guides, with topics exploring the perceived acceptability, feasibility, and impact of the EASE intervention in North Lebanon, including facilitators and barriers of implementation and recommendations for improvements.

### Analysis

#### Quantitative analysis

Descriptive statistics (means, standard deviations, N’s, percentages) were used to explore baseline demographic characteristics. Cronbach’s alpha was used to evaluate the internal reliability of outcome measures at baseline.

We explored the difference in change in each outcome between the EASE and ETAU groups from baseline to each follow-up using linear mixed effects regression models. Models included fixed effects of treatment arm (1 = EASE; 0 = ETAU), time (0 = T0; 1 = T1; 2 = T2), and interaction terms of treatment arm X time. Random effects included participant ID, EASE group ID (for treatment group), and family ID, which was necessary to account for the fact that there were several pairs of siblings included in the analysis. For each outcome, we present the model-predicted means and corresponding 95% confidence intervals for each treatment arm and time point. We do not present the ‘treatment effect’ or *p*-values as this feasibility study was not powered to detect statistically significant treatment effects.

Models were each estimated three times for each outcome. First, following an intent to treat principle, we estimated models including all study participants at all timepoints. Participants were analysed according to their randomization allocation and mean imputation was used if a variable was missing or the participant missed a follow-up visit. Second, we estimated the models with participants analysed according to the group they were randomized to but did not conduct imputation; therefore, not all participants contributed follow-up data. Third, we estimated the models following imputation but only included a subset of the EASE adolescent participants who were defined as ‘treatment completers’ (defined as having completed five or more sessions). Analyses were conducted using Stata, version 15.

#### Qualitative* analysis*

Qualitative data was analysed using inductive and deductive thematic techniques, following the framework method [41]. After familiarization with the data and open-coding, an analytical framework was agreed and applied by two authors (KT and AG), with adjustments made until inter-rater reliability was achieved, and the framework was considered to capture all pertinent themes in the data. The framework matrices were explored to identify associations between the themes, triangulate between sub-groups, and ensure there was sufficient evidence to explain the findings.

## Results

### Sample characteristics

Table 1 provides characteristics of the adolescent study sample at baseline. Forty-five percent of the sample were female and the average age was 11.7 (*SD* = 1.3). Only two participants were of Lebanese nationality, with the remainder of Syrian nationality and the majority born in Syria. Table 2 provides characteristics of the caregiver study sample at baseline. The average age of accompanying caregivers was 38.4 years (*SD* = 7.8); 72% were mothers, 22% fathers, and 6% other family members. Households had an average of more than five children per family, and all households had an average income of less than $600 per month (which at the time of this study was equivalent to 900,000 Lebanese pounds, prior to later devaluation). The vast majority of caregivers had less than high-school level education, and very few had salaried employment. Caregivers reported high rates of adolescent exposure to potentially traumatic events, with an average of five events per adolescent.

### Aim 1: Recruitment, screening, completion, and retention rates

Participants were enrolled between September 2018 and January 2019. We faced challenges with recruitment, with the most common reasons reported by outreach team being: (i) lack of interest in psychosocial support with preference for financial or education support; (ii) difficulty attending due to adolescents’ work or school schedule. It was difficult to recruit participants from outside the existing War Child programmes, since a trusting relationship with these families was not built yet. Many Lebanese families declined participation due to the perception that the intervention predominantly targeted Syrians, illustrating tensions between the communities. In order to proceed with delivering interventions for those enrolled within one month from completing baseline, we conducted implementation of EASE groups in two waves, with the first groups commencing sessions while recruitment for the next groups took place.

**Fig. 1:**
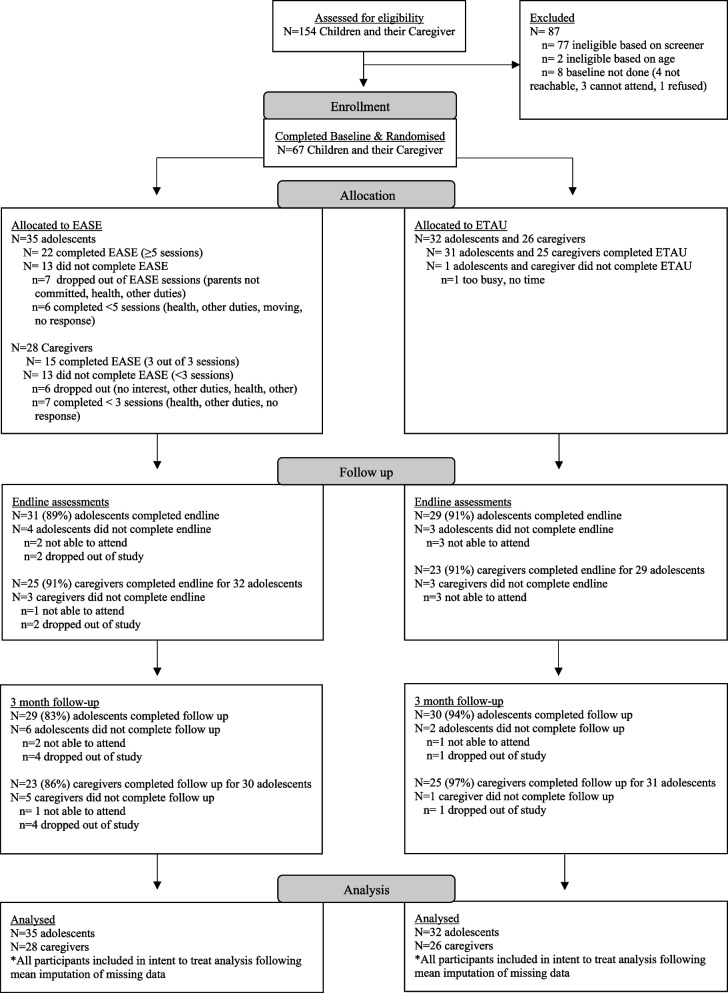
CONSORT Flow Chart

We registered 266 adolescents as interested in the study, of which only 154 (58%) completed screening assessments. At screening, 75 were eligible (49%), 77 were not eligible due to not meeting the cut-off on CPDS and 2 were not eligible due to disability. Of those eligible, 67 (89%) completed baseline, 35 were randomized to EASE and 32 were randomized to ETAU (see Fig. 1). To accommodate delays in outreach and scheduling challenges, we conducted five EASE groups, three for males and two for females. There were five pairs of siblings and one trio of siblings in the EASE arm, and six pairs of siblings in the ETAU arm. Twenty-eight caregivers of EASE adolescents and twenty-six caregivers of ETAU participated in the study. Participating adolescents had an average CPDS screening score of 6.9 (*SD* = 1.6) in the EASE group, and 6.1 (*SD* = 1.2) in the ETAU group.

**Fig. 2:**
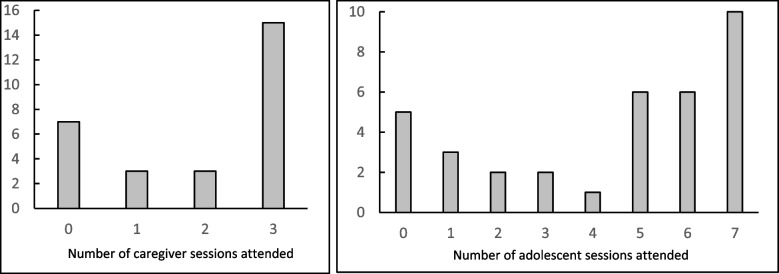
Attendance at EASE sessions for adolescents and caregivers

Only one family did not complete the ETAU home visit session (97% completed). Conversely, only 22 (63%) adolescents completed at least five sessions of EASE, which was specified as treatment completion, and only 15 (54%) of caregivers completed the three caregiver sessions. Attendance at total number of EASE sessions for adolescents and caregivers is illustrated in Fig. 2; average adolescent sessions attended was 4.37, no systematic patterns in attendance at different sessions were observed, and the most common reasons for missing sessions were attending work, and illness. Retention in T1 assessments was 89% for EASE and 91% for ETAU adolescents, and retention in T2 assessments was 83% for EASE and 94% for ETAU adolescents. Analysis of drop-out and lost to follow up did not suggest any significant demographic predictors of missing an assessment.

### Aim 2: Feasibility and acceptability of EASE delivered by trained non-specialists

#### Fidelity and competency

*EASE Sessions.* According to facilitator checklists, most adolescent sessions took longer than the allocated time (range 90 to 135 min; average 110 min) with some sessions taking longer due to managing behavioural challenges. The majority (80%) of adolescent session components were completed across all groups. Only eight components were not completed- four were reviewing prior session, one was reviewing homework and three were other activities not implemented due to time. Caregiver sessions took on average 106 min (range 90 to 120 min), and only two session components were not implemented across all groups: one welcome activity, and one role play activity.

Five session observations were able to be completed (8.3% of EASE sessions), and these indicated that only 1 session component (homework review) was omitted in 1 session, 82% of components were delivered ‘well’, 18% were delivered ‘partially well’, and none were delivered ‘poorly’. Of the four general competencies rated in each of the five sessions, 15 (75%) were done well, 1 (5%) was done partially well, and 4 (20%) were not applicable in that session (i.e. responding to distress, or safety management).

*ETAU Sessions.* According to facilitator session checklists, ETAU sessions ranged between 16 and 35 min (*M* = 26 min), with the exception of one session that lasted 1 h 35 min. All components were marked as completed. Three sessions (10%) were observed, with 7 components (78%) rated as being delivered well and, 2 components (22%) rated as needing improvement.

#### Qualitative findings

The qualitative analysis identified five overarching themes: (i) Overall positive experiences of EASE; (ii) Acceptability and feasibility of intervention components; (iii) Impact of EASE on adolescents and caregivers; (iv) Facilitators and barriers to participant adherence and attendance; (v) Scale-up and integration into community. There was positive feedback about the intervention and the impact it had on adolescent behaviour and emotions, and relationships in the family and outside. Participants reported that adolescents benefited directly from EASE strategies as well as increased support from caregivers. One boy reported improvements in his own behaviour: *“After I went to the [centre] and I see people fighting I am careful not to fight with anyone. So the [intervention] helped us a lot. A lot has changed with me.” (*male adolescent, 12 years old*).*

Intervention content was generally reported to be understandable, relevant, and useful. The strategies of slow breathing, problem solving and noticing strengths were most positively received. Some challenges were noted with understanding the psychoeducation about the vicious cycle of inactivity, and related activity planning / behavioural activation strategies. Facilitators and trainers noted that repetition of content helped with learning, but was also a source of boredom. Participants reported that there was a lack of interactive activities, and the storybook, which forms the core of the sessions, was not engaging enough for adolescents in this study. Disruptive behaviour in sessions was common and was a reported concern for facilitators and for other adolescents, who felt it disturbed their experience. Trainers and facilitators reported that the intervention often assumed adolescents and caregivers could read and write, while this was commonly not the case. Furthermore, caregiver sessions were perceived as too dense and long.

There was some dissatisfaction among caregivers that ‘*taboo*’ topics were discussed with adolescents. Caregivers did not like that adolescents were asked during screening about suicidality, fearing that this would increase likelihood of suicidality amongst adolescents. They also mistakenly often believed that adolescents were being asked questions about prior experiences of traumatic events, since caregivers were asked to complete the trauma inventory for their adolescent. Finally, it was evident that there were some misunderstandings among adolescents and caregivers about the nature of confidentiality in the intervention. An emphasis had been placed on the confidentiality of what was discussed in sessions, and adolescents and caregivers were told that personal disclosures discussed in the adolescent groups would not be shared with caregivers, except where required for safety, and vice versa. However, many adolescents and caregivers believed that adolescents were not allowed to discuss the content of their sessions with their caregivers, counter to the aims of the intervention.

Outreach and engagement were noted to be challenging, with one trainer mentioning: “*the moment they [caregivers] are coming, they are being loyal to the intervention, they are not dropping out. The problem is the motivation to start, here lies the big problem, not in the follow-up*.” Different times of the year brought different challenges for engagement of families in the intervention due to competing priorities for securing livelihood or educational opportunities, including seasonal agricultural work, or school enrolment periods. Attendance often suffered due to illness or competing work responsibilities either inside or outside the home, illustrated by the case of one EASE participant who said of his employer: *“They don’t let me take days off. Also, our situation is hard, I am spending money on my parents, and there is work, I cannot go, I mean they don’t let me take days off.”* (Male adolescent, 14 years old). While transport was provided, either in terms of a bus for adolescents, or reimbursement of travel costs for caregivers, there were issues with both of these options. Caregivers were reluctant for their adolescents to travel long distances away from home on a bus, and when receiving reimbursement for travel expenses they often needed to take a loan from someone to cover the initial outlay.

Facilitators reported that the training was comprehensive and appreciated the active role-plays and feedback, along with anticipating and learning how to respond to challenging situations. They reported feeling overwhelmed with the amount of new content at first, but soon mastered it through implementation. Supervisors noted that additional support was required in managing difficult in-session behaviour, explaining complex strategies to participants to enable application to their own situation, and presenting strategies in engaging ways for adolescents. Results indicated that EASE facilitators were deemed acceptable delivery agents and perceived as competent by all stakeholder groups. Trainers attributed this success to 1) training and supervision were comprehensive; 2) recruited facilitators had significant experience prior to training in EASE; 3) a supportive and sharing environment between team members. Additionally, facilitators cited multiple motivations to stay in the intervention, in contrast to previous task-shifting interventions where low facilitator motivation has been a barrier to implementation. While financial incentives were mentioned, non-financial incentives such as the promise of a certificate, personal skill development, willingness to help, and perceived value of the intervention were all important factors. After several rounds of implementation, supervision sessions were perceived as being repetitive and there was a recommendation to make them more active.

### Aim 3: Feasibility and psychometric properties of outcome measures and trends over time

Internal reliability, means and standard deviations at T0, T1, and T2 for each outcome measure are presented in Tables 3 and 4. Item-level missing data on outcome measures was low (< 10% for all but eight questionnaire items). For the eight items with > 10% missing, missingness was attributable to responses of ‘*don’t know’*, or ‘*not applicable’*. Five of these items related to experiences at school, and high rates of missingness were explained by lack of school attendance. Internal reliability was good for all adolescent-report measures (ranging from 0.77 to 0.83), and caregiver reported PSC (α = 0.87) and the K6 (α = 0.80). On subscales of the Alabama Parenting measure it ranged from 0.49 for inconsistent parenting, to 0.79 for positive parenting. Internal consistency was generally poor on adolescent-report subscales for PSC-attention (α = 0.43), PSC-Internalizing (α = 0.67), PSC- Externalizing (α = 0.57), CRIES-Intrusion (α = 0.27), and CRIES-Avoidance (α = 0.59). For caregiver-reported PSC subscales, similar findings were seen on PSC-Attention (α = 0.53), PSC-Internalizing (α = 0.61), and PSC-Externalizing (α = 0.57). Further analyses on these subscales are therefore not reported here.

**Table 3:** Means and effect sizes for child outcomes

Outcome (alpha)		Mean (95% CI)						
	Baseline			Endline			3-month follow-up	
	EASE (*N* = 35)	ETAU (*N* = 32)	EASE (*N* = 35)	ETAU (*N* = 32)	d	EASE (*N* = 35)	ETAU (*N* = 32)	d
PSC (α = 0.78)	20.4 (17.2, 23.5)	21.0 (17.0, 25.0)	21.0 (17.8, 24.2)	20.7 (16.7, 24.6)	0.12	21.3 (18.2, 24.5)	20.3 (16.3, 24.2)	0.21
PHQ (α = 0.77)	5.2 (3.4, 7.0)	5.4 (3.1, 7.7)	5.3 (3.5, 7.1)	5.1 (2.8, 7.4)	0.10	5.0 (3.2, 6.8)	5.6 (3.3, 7.9)	-0.08
CRIES (α = 0.78)	21.9 (17.0, 26.7)	20.9 (15.0, 26.8)	22.7 (17.8, 27.5)	20.8 (15.0, 26.7)	0.07	19.2 (14.3, 24.0)	23.9 (18.0, 29.8)	-0.44
Functioning (α = 0.79)	6.4 (4.0, 8.8)	6.9 (3.8, 10.0)	5.8 (3.4 8.2)	7.4 (4.3, 10.5)	-0.18	6.7 (4.3, 9.1)	6.1 (2.9, 9.2)	0.18
Wellbeing^ (α = 0.83)	46.3 (43.0, 49.6)	46.4 (43.0, 49.8)	48.8 (45.6, 52.1)	49.9 (46.5, 53.3)	-0.10	43.6 (40.3, 46.9)	44.1 (40.7, 47.6)	-0.05

Means, SDs are based on coefficients and combination of coefficients from mixed effects model

Cohen’s d effect size was calculated by dividing the predicted difference in mean change from the mixed effects model by the pooled, imputed baseline SD. Negative effect size indicates a larger change in EASE compared to ETAU

Model included fixed effects of arm, time and arm X time interaction, and random effects of pt_code, siblingid, and Ease group

All participants were included in the model at all timepoints following mean imputation

^ For this measure, an increased score indicates an improvement. For other measures, decreased scores indicate reduction in symptoms/impairment

**Table 4:** Means and effect sizes for caregiver outcomes

Outcome (alpha)	Mean (95% CI)							
	Baseline		Endline			3-month follow-up		
	EASE (*N* = 35)	ETAU (*N* = 32)	EASE (*N* = 35)	ETAU (*N* = 32)	d	EASE (*N* = 35)	ETAU (*N* = 32)	d
PSC (α = 0.87)	19.3 (14.5, 24.1)	24.1 (17.6, 30.7)	20.6 (15.8, 25.4)	26.0 (19.4, 35.6)	-0.06	20.8 (16.0, 25.6)	22.9 (20.4, 33.5)	-0.13
K6* (α = 0.80)	20.8 (18.8, 22.8)	20.3 (18.2, 22.4)	20.0 (18.0, 22.0)	21.5 (19.4, 23.6)	-0.34	20.9 (18.8, 22.9)	19.9 (17.8, 22.0)	0.07
Alabama-Involvement*^ (α = 0.78)	31.0 (28.3, 33.7)	32.0 (29.2, 34.8)	34.4 (31.7, 37.1)	35.2 (32.4, 38.0)	0.02	31.7 (29.0, 34.3)	31.3 (28.5, 34.1)	0.17
Alabama-Positive*^ (α = 0.79)	22.8 (21.0, 24.5)	22.9 (21.0, 24.7)	23.6 (21.8, 25.3)	23.8 (22.0, 25.6)	-0.01	21.9 (20.1, 23.6)	22.0 (20.2, 23.9)	< 0.01
Alabama-Monitoring* (α = 0.69)	16.3 (14.2, 18.4)	16.0 (13.5, 18.5)	18.4 (16.3, 20.5)	19.1 (16.6, 21.6)	-0.17	15.2 (13.0, 17.3)	14.7 (12.2, 17.2)	0.03
Alabama-Inconsistent* (α = 0.49)	15.4 (14.0, 16.8)	17.2 (15.7, 18.6)	16.2 (14.8, 17.6)	17.5 (16.1, 19.0)	0.10	14.9 (13.5, 16.3)	17.4 (15.9, 18.8)	-0.17
Alabama-Corporal* (α = 0.65)	7.7 (6.7, 8.6)	8.0 (7.0, 9.0)	7.3 (6.3, 8.2)	7.5 (6.5, 8.5)	0.01	7.7 (6.8, 8.7)	8.1 (7.1, 9.1)	-0.02

Means, SDs are based on coefficients and combination of coefficients from mixed effects model

Cohen’s d effect size was calculated by dividing the predicted difference in mean change from the mixed effects model by the pooled, imputed baseline SD. Negative effect size indicates a larger change in EASE compared to ETAU

Model included fixed effects of arm, time and arm X time interaction, and random effects of pt_code, siblingid, and Ease group

All participants were included in the model at all timepoints following mean imputation

^*^Caregiver responds only once regardless of how many children they have in the study so observations are fewer: (EASE *N* = 28; ETAU *N* = 26)

^ For this measure, an increased score indicates an improvement. For other measures, decreased scores indicate reduction in symptoms/impairment

Baseline levels of distress on outcome measures were relatively low overall. Previously we proposed a cut-off of 21 on the adolescent-report PSC-35 (Brown F, Steen F, Taha K, Koppenol-Gonzalez GV, Aoun M, Bryant RA, et al: Validation of Arabic versions of the Child Psychosocial Distress Screener and Pediatric Symptom Checklist for young adolescents living in vulnerable communities in Lebanon, Under review), while our sample had an average score of < 21 at baseline. Similarly, the recommended cut-off for the PHQ-9 is 11 [35], while our sample had an average score of only 5.3 at baseline. In exploratory models looking at trends over time, model results were similar for all three analysis approaches (ITT with mean imputation; ITT without mean imputation; per protocol with only EASE adolescent treatment completers), therefore we present only the ITT with mean imputation here. As can be seen in Tables 3 and 4, for all outcomes, there was negligible mean change from baseline to follow-up and no meaningful apparent difference in mean change between the treatment groups.

Several challenges were noted with particular items on assessments. For the assessors and participants, the items with negatively phrased statements were challenging, and additional prompts were added to ensure understanding. For these and other items that were persistently challenging, warnings were added in the Kobo form for assessors to pay close attention. Improvements to the Arabic translations of some items were also recommended and implemented after the feasibility study.

### Aim 4: Safety and feasibility of trial procedures

#### Randomisation

Randomisation resulted in approximately equal group sizes, and there were no meaningful differences in demographic characteristics between the groups. There were no indications of acceptability issues with the procedure, and interventions were delivered as allocated.

#### Contamination

Overall, reliability of contamination data was questionable due to participant responses frequently not indicating adequate recall of services received. At the three-month follow-up, approximately half of the EASE participants reported sharing information learned in the sessions with others, however these people were not in the ETAU group. Approximately 19% of the ETAU participants reported having seen or heard about EASE materials, but upon further enquiry the information cited did not appear to be from the EASE intervention in around half of these cases. The hotline list was reportedly not used by many participants, and those who did use it did not find it helpful.

#### Blinding

There was only one instance of reported unblinding during the assessments, where an adolescent exposed their allocation at the start of an interview, after which the assessor directly informed the research coordinator who assigned a new assessor as per protocol. Only 41% of assessor allocation guesses were correct at T1 and 53% were correct at T2, indicating that blinding was satisfactorily maintained.

#### Adverse events

One SAE was reported (physical abuse of an assessor by an adolescent participant), and ten adverse events were reported (thoughts of suicide by the adolescent, or disclosure of physical, sexual, or emotional abuse, neglect, or exploitation). Incidents were reported as per protocol, and DSMC reviews indicated that these were not perceived to be linked to the study or intervention. Referrals were made to additional services as needed to ensure wellbeing and safety of staff and participants.

## Discussion

The overall aim of this randomized feasibility study of EASE in Lebanon was to inform adaptations needed to intervention and research protocols for a fully-powered RCT to assess effectiveness. We noted challenges with outreach and saw significant attrition between registration of interest and screening time-points, and between screening and baseline for those eligible. There were challenges among the EASE participants in terms of regular attendance at sessions, with only 63% of adolescents completing five or more EASE sessions; common reasons cited were school and work commitments, or transport issues. Nonetheless we found good retention rates among adolescents and caregivers in completing endline and follow-up assessments. Randomisation and blinding procedures were effective and feasible, and we noted minimal contamination. Fidelity and competency of EASE implementation was satisfactory, though time management and behaviour management were issues in some sessions. Outcome measures showed good psychometric properties on total scale scores, with low item-level missing data for most questionnaires. Our screening measure resulted in a positive screening rate of 49%, while baseline levels of distress on outcome measures were relatively low. We did not identify substantial patterns of change overtime on our outcome measures, (between or within groups) despite qualitative reports of positive impacts of EASE. We responded to a high number of adverse events throughout the study, necessitating referrals to other services, though none were deemed to be caused by or linked to participation in the study.

Findings of this feasibility trial resulted in several improvements to study procedures to inform a forthcoming larger definitive trial [24]. Firstly, recommendations from this feasibility study (along with concurrent studies conducted in Jordan [23] and Tanzania [26]), lead WHO developers to make the following modifications to the EASE intervention: (i) more interactive activities were added to adolescent sessions, (ii) the storybook used in adolescent sessions was re-written by a creative writer to enhance engagement, (iii) caregiver sessions were shortened and revised to reduce the need for literacy. For the Lebanon context specifically, guidance was added to hold the first caregiver session prior to the first adolescent session, to allow a clearer explanation to caregivers about the content of the adolescent sessions including confidentiality expectations, and the extent to which ‘taboo’ topics such as suicide and traumatic events will be discussed. Furthermore, additional facilitator training was provided on behaviour management, engaging the group, and showing empathy for challenges raised, and avoiding giving advice. Supervision sessions were adapted to be more active and skills based.

RCT procedures were adapted to overcome transportation challenges: (i) group sessions were held as close as possible to homes; (ii) caregivers were provided with the choice between provision of transportation via bus, or reimbursement of travel costs; (iii) outreach teams improved communication with caregivers before and during adolescent transportation; (iv) improvements were made to the contamination tool and assessor training in order to improve reliability of this measurement. We added an explicit check at screening that both adolescents and caregivers were willing to commit to attend to all EASE sessions should they be allocated to that condition. Recommendations were made to enable flexible scheduling of sessions to include evenings and weekends, and have facilitators remain in contact with caregivers over WhatsApp. Given the intense outreach efforts required to reach our pilot sample, we employed additional outreach staff for the RCT, and provided substantive training on methods for explaining the intervention, responding to concerns, and generating interest. To respond to the high rates of child protection and acute mental health needs experienced in the feasibility study, we hired additional staff members to support on these aspects during the full trial to ensure safety and adequate referral and follow up. The suitability of these adjustments will be monitored in the full trial.

While most outcome measures were deemed understandable and appropriate, we adjusted the wording of some items that were found to be challenging and removed non-endorsed traumatic events from the trauma inventory. Notably, the CPDS screening tool lead to a sample with relatively low levels of distress overall on outcome measures, despite a prior validation study (Brown F, Steen F, Taha K, Koppenol-Gonzalez GV, Aoun M, Bryant RA, et al: Validation of Arabic versions of the Child Psychosocial Distress Screener and Pediatric Symptom Checklist for young adolescents living in vulnerable communities in Lebanon, Under review). One possibility for the lack of change over time in our study is a floor effect, possibly due to the fact that the CPDS assesses presence of psychosocial risk factors, rather than psychological distress. Based on this, we changed our screening tool for the RCT to the PSC-17 tool. While the increased length adds challenges for population-level screening, it has shown good internal consistency, test–retest reliability, and concurrent validity against a gold-standard psychiatrist assessment in Lebanon (Brown F, Steen F, Taha K, Koppenol-Gonzalez GV, Aoun M, Bryant RA, et al: Validation of Arabic versions of the Child Psychosocial Distress Screener and Pediatric Symptom Checklist for young adolescents living in vulnerable communities in Lebanon, Under review). Given the poor psychometric properties of PSC and CRIES subscales in this sample, our primary hypotheses for the full trial will focus on total scale scores.

We expect that these amendments will lead to improved implementation of the EASE intervention and trial procedures for the RCT. A simultaneous feasibility trial of EASE in Jordan found similar lack of movement on outcomes, and similar possible floor effects [23]. This sample was similar in terms of past traumatic events experienced, although there were no adverse events reported throughout this study, more adolescents were currently attending school, and less were working to provide income for the family. Better attendance was observed, which might be explained by less disruptive circumstances. Another study of a non-specialist psychological intervention in Lebanon conducted during the same period demonstrated similar difficulties with engagement [42], suggesting that pervasive stressors and community dynamics in Lebanon may influence demand-side factors in delivering psychological care.

Although we have made significant adjustments to our implementation plan for the RCT to promote smooth implementation, these adjustments predominantly involve mobilising additional resources in terms of outreach staff and activities, support for following up with significant child protection and mental health needs, and enhanced transport options to sessions. While these resources may be needed in order to ensure adequate and comprehensive care is available for those who need it, it is important to note for future implementation of such interventions in protracted crises that these interventions are unlikely to be viable as standalone programmes, and must be integrated within existing services and support structures in order for them to be sustainable and effective. EASE may be optimally positioned within a multi-level and multi-sectoral care system whereby adolescents may be engaged in community level psychosocial support activities and educational activities, through which identification and referral to EASE can be made, and through which direct referrals can be available to more intensive case management for protection concerns, or specialised services for more severe mental health concerns, as required [43]. In addition, it will be important that attention also turns to community engagement activities, including mechanisms to sensitise communities to mental health activities, and reduce stigma around accessing such interventions.

While the group format of EASE has advantages in terms of increasing numbers of adolescents reached, and promoting social support, the scheduling of group session times and coordinating common locations reachable by adolescents across a fairly broad geographical area presented significant challenges for attendance, and necessitated significant transport costs. Future research should compare the effectiveness, overall cost-effectiveness, and sustainability of individual versus group treatment models of EASE.

### Limitations

The small sample size, though intentional, prevents us from conducting meaningful subgroup analyses to understand whether EASE may show trends in change overtime for certain demographic groups, or in situations of higher adolescent and caregiver participation in the intervention. This will be explored in a forthcoming fully-powered RCT. This feasibility trial was registered retrospectively which limits the ability to guarantee non-selective outcome reporting. The full RCT has been registered prospectively. Furthermore, the applicability and impact of the EASE intervention may have been under-estimated due to a sample that overall was not scoring high on distress at baseline. Intervention costs and health economic analyses are important in definitive trials to estimate cost-effectiveness of interventions, and feasibility of collecting relevant data could have been piloted in this study. Lastly, while we conducted follow up interviews with drop-outs from the intervention to understand the reasons, relatively little is known about the perceptions and barriers for engagement of those community members who we approached but declined to participate. Understanding these experiences would be helpful to inform outreach and engagement activities and inform future service delivery.

## Conclusions

Our findings indicate that the EASE intervention and study procedures are overall safe and acceptable for adolescents and caregivers in North Lebanon. High levels of adversity and competing demands led to challenges with engagement and attendance in the intervention, and many referrals to other services were needed throughout the intervention. This feasibility study allowed identification of several improvements to the EASE intervention and trial procedures to enhance effectiveness and implementation feasibility. The forthcoming fully powered trial will assess the impact of EASE in this population.

## Data Availability

The datasets used during the current study are available from the corresponding authors on reasonable request.

## Abbreviations

APQ: Alabama parenting questionnaire
CPDS: Child psychosocial distress screener
CRIES: Children’s revised impact of events scale
DSMC: Data safety management committee
EASE: Early Adolescent Skills for Emotions
ETAU: Enhanced treatment as usual
K6: Kessler 6
LMIC: Low and middle income country
RCT: Randomized controlled trial
PHQ: Patient Health Questionnaire
PSC: Pediatric Symptom Checklist
(S)AE: (Serious) adverse events
WEMWBS: Warwick Edinburgh Mental Wellbeing Scale
WHO: World Health Organization
